# Neuroprotective activity of ursodeoxycholic acid in *CHMP2B*^*Intron5*^ models of frontotemporal dementia

**DOI:** 10.1016/j.nbd.2020.105047

**Published:** 2020-10

**Authors:** Ryan J.H. West, Chris Ugbode, Laura Fort-Aznar, Sean T. Sweeney

**Affiliations:** a Department of Biology, University of York, York YO10 5DD, UK; b Sheffield Institute for Translational Neuroscience, University of Sheffield, S10 2HQ, UK; c Neuroscience Institute, University of Sheffield, Western Bank, Sheffield S10 2TN, UK

**Keywords:** Amyotrophic Lateral Sclerosis, ALS, CHMP2B, Frontotemporal dementia, FTD, UDCA, Glutathione, MND, Neurodegeneration

## Abstract

Frontotemporal dementia (FTD) is one of the most prevalent forms of early-onset dementia. It represents part of the FTD-Amyotrophic Lateral Sclerosis (ALS) spectrum, a continuum of genetically and pathologically overlapping disorders. FTD-causing mutations in *CHMP2B*, a gene encoding a core component of the heteromeric ESCRT-III Complex, lead to perturbed endosomal-lysosomal and autophagic trafficking with impaired proteostasis. While *CHMP2B* mutations are rare, dysfunctional endosomal-lysosomal signalling is common across the FTD-ALS spectrum. Using our established *Drosophila* and mammalian models of CHMP2B^Intron5^ induced FTD we demonstrate that the FDA-approved compound Ursodeoxycholic Acid (UDCA) conveys neuroprotection, downstream of endosomal-lysosomal dysfunction in both *Drosophila* and primary mammalian neurons. UDCA exhibited a dose dependent rescue of neuronal structure and function in *Drosophila* pan-neuronally expressing CHMP2B^Intron5^. Rescue of CHMP2B^Intron5^ dependent dendritic collapse and apoptosis with UDCA in rat primary neurons was also observed. UDCA failed to ameliorate aberrant accumulation of endosomal and autophagic organelles or ubiquitinated neuronal inclusions in both models. We demonstrate the neuroprotective activity of UDCA downstream of endosomal-lysosomal and autophagic dysfunction, delineating the molecular mode of action of UDCA and highlighting its potential as a therapeutic for the treatment of FTD-ALS spectrum disorders.

## Highlights

• Defective endosomal proteostasis is a hallmark of CHMP2B^Intron5^ FTD/ALS.
• CHMP2B^Intron5^ causes neuronal dysfunction in *Drosophila* and mammalian models.
• FDA approved compound UDCA rescues neuronal dysfunction in both models.
• UDCA treatment does not rescue endosome function or excess ubiquitinated protein.
• UDCA treatment highlights glutathione as a novel regulator of CHMP2B^Intron5^ pathology.

## 1 Introduction

Frontotemporal Dementia (FTD) is a common cause of early-onset dementia, second only to Alzheimer's Disease, with a typical age of onset under 65 years of age. FTD is commonly used as an umbrella term referring to a genetically, pathologically and clinically heterogeneous group of neurodegenerative disorders associated with Frontotemporal lobar degeneration (FTLD), a progressive atrophy of the frontal and temporal cortices. These include behavioural variant FTD (bvFTD), primary progressive aphasia, semantic dementia and FTD with Amyotrophic Lateral Sclerosis (ALS). Of these, bvFTD is the most prevalent, accounting for ~60% of all cases. FTD represents a significant societal and medical challenge with no current effective treatment or cure. Nearly half of all cases of FTD have a familial precedent, indicating a genetic cause or predisposition. FTD loci collectively representing ~40% of all FTD cases reveal a clinical, genetic and pathological overlap with ALS (Ling et al., 2013). Mutations in *TAR DNA-binding protein* (TARDBP) (Borroni et al., 2009; Kovacs et al., 2009; Sreedharan et al., 2008; Van Deerlin et al., 2008), *Fused in Sarcoma* (FUS) (Kwiatkowski Jr. et al., 2009; Vance et al., 2009), *C9ORF72* (DeJesus-Hernandez et al., 2011; Gijselinck et al., 2012; Renton et al., 2011), *Ubiquilin-2*(Deng et al., 2011), *p62/sequestosome-1*(Fecto et al., 2011*;*Rubino et al., 2012*;*Teyssou et al., 2013), *Valosin containing Peptide* (VCP) (Johnson et al., 2010; Watts et al., 2004) *Charged Multivesicular Body protein 2B* (*CHMP2B*) (Parkinson et al., 2006; Skibinski et al., 2005) and more recently *TANK Binding Kinase 1* (*TBK1*) (Freischmidt et al., 2015*;*Pottier et al., 2015*)* can be causative for ALS or FTD, or give rise to a disease that has clinical characteristics of both conditions in the same individual (Ling et al., 2013). This genetic and pathological continuum suggests common or partially shared disease mechanisms for this class of FTD-ALS.

In a number of previous studies, we have established *Drosophila* and primary mammalian neuron models of FTD associated with the bvFTD-disease causing *CHMP2B*^*Intron5*^ mutation (Ahmad et al., 2009; West et al., 2015; West et al., 2018). CHMP2B encodes a core subunit of the endosomal sorting complex required for transport-III (ESCRT-III), a fundamental component of the ESCRT machinery involved in the biogenesis of multivesicular bodies (MVB) in the endosomal-lysosomal trafficking pathway. The *CHMP2B*^*Intron5*^ mutation results in a C-terminal truncation of the protein removing the microtubule-interacting and transport (MIT)- interacting motif (MIM) domain and the ability to associate with Vps4, the ATPase known to dissociate the ESCRT-III complex (Stuchell-Brereton et al., 2007). The *CHMP2B*^*Intron5*^ mutation results in significant perturbations to endosomal-lysosomal (Urwin et al., 2010; van der Zee et al., 2008) and autophagosomal trafficking in post-mortem tissue and mice expressing this truncated protein (Filimonenko et al., 2007; Ghazi-Noori et al., 2012; Lee et al., 2007). Other mutations in *CHMP2B* have been identified in FTD and ALS patients (Cannon et al., 2006; Cox et al., 2010; Ferrari et al., 2010; Ghanim et al., 2010; Momeni et al., 2006; Parkinson et al., 2006; Rizzu et al., 2006; van Blitterswijk et al., 2012; van der Zee et al., 2008) and similar endosomal disruptions have been observed in associated patient tissue and rat primary neurons expressing these *CHMP2B* mutations (Cox et al., 2010; Han et al., 2012; Lee et al., 2007). Expression of CHMP2B^Intron5^ results in an unregulated synaptic growth phenotype at the *Drosophila* third instar larval neuromuscular junction (NMJ) associated with endosomal perturbation and activated JNK signalling (West et al., 2015; West et al., 2018). Endosomal disruption and activated JNK signalling also results in a dendritic retraction phenotype in primary mammalian neurons transfected with CHMP2B^Intron5^ (West et al., 2018).

Clinical tests have identified the FDA approved compound TUDCA (tauro-ursodeoxycholic acid), a precursor of the bile acid UDCA (ursodeoxycholic acid) as a potential treatment for ALS (Elia et al., 2016; Parry et al., 2016; Parry et al., 2010). UDCA is an established treatment for primary biliary cirrhosis and is well tolerated in humans with reasonable penetration of the blood brain barrier (Parry et al., 2010;Jazrawi et al., 1994). UDCA has been shown to exhibit both anti-apoptotic and anti-autophagic activity in various cell types (Amaral et al., 2009), however a defined molecular mode of action has yet to be established. Having previously identified both autophagic and apoptotic pathways to be perturbed in CHMP2B^Intron5^ models of FTD (Ahmad et al., 2009; Lee et al., 2007; Lu et al., 2013; West et al., 2015; West et al., 2018) we asked whether UDCA could alleviate pathological CHMP2B^Intron5^ driven phenotypes in both *Drosophila* and mammalian models of FTD. We demonstrate that the administration of UDCA is sufficient to alleviate neuronal aberrations in both *Drosophila* and mammalian primary neuron models of bvFTD associated with the *CHMP2B*^*Intron5*^ mutation. UDCA rescued elevated apoptotic cascades downstream of autophagic and endosomal perturbations. In addition, UDCA administration allowed us to identify a role for Glutamate-Cysteine Ligase Catalytic Subunit (GCLC) in alleviating dysregulated neuronal phenotypes in CHMP2B^Intron5^ models. This work identifies GCLC as a novel regulator of CHMP2B^Intron5^ driven pathology and provides insight into the neuroprotective activity of UDCA, acting downstream of endosomal-autophagic perturbations.

## 2 Results

### 2.1 UDCA and UCA exhibit a dose dependent rescue of neuronal perturbations in *CHMP2B*^*Intron5*^ expressing *Drosophila*

**Fig. 1:**
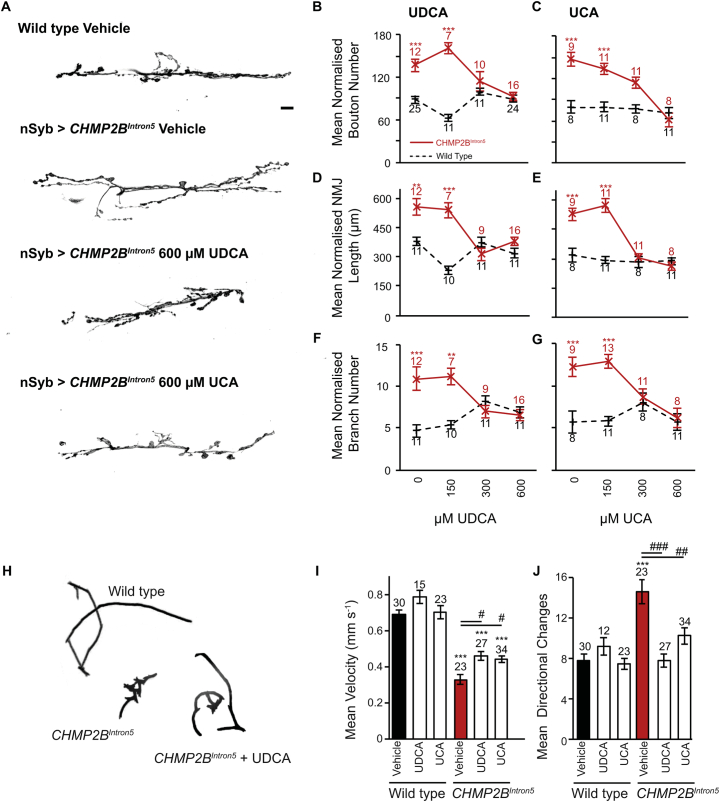
UDCA and UCA Exhibit a Dose Dependent Rescue of Synaptic Overgrowth at the *Drosophila* NMJ. A. Representative micrographs showing synaptic overgrowth at the *Drosophila* third instar wandering larval NMJ (Muscle 6/7, hemisegment A3) in larvae pan-neuronally expressing (*nSyb*-Gal4) UAS-CHMP2B^Intron5^ administered vehicle, UDCA or UCA. Scale bar = 10 μm. B—C. Quantification of normalised bouton number in wild type (dashed black line) and CHMP2B^Intron5^ expressing (*nSyb*-Gal4) (solid red line) 3rd instar larvae in response to increasing doses of UDCA (B) and UCA (C). ANOVA with post-hoc Tukey comparison to vehicle treated (0 μM) control ^⁎⁎⁎^p < .001. D, E. Quantification of normalised NMJ length in wild type (dashed black line) and CHMP2B^Intron5^ expressing (*nSyb*-Gal4) (solid red line) 3rd instar larvae in response to increasing doses of UDCA (D) and UCA (E). ANOVA with post-hoc Tukey comparison to vehicle treated (0 μM) control ^⁎⁎^p < .01, ^⁎⁎⁎^p < .001. F, G. Quantification of normalised branch number in wild type (dashed black line) and CHMP2B^Intron5^ expressing (*nSyb*-Gal4) (solid red line) 3rd instar larvae in response to increasing doses of UDCA (F) and UCA (G). ANOVA with post-hoc Tukey comparison to vehicle treated (0 μM) control ^⁎⁎^p < .01, ^⁎⁎⁎^p < .001. H–J. Addition of UDCA or UCA to *Drosophila* food alleviates aberrant crawling behaviour in 3rd instar wandering larvae pan-neuronally (*nSyb*-Gal4) expressing CHMP2B^Intron5^. H. Representative traces of crawling path. I. Median crawling speed. J. Mean number of directional changes. ANOVA with post-hoc Dunnett's comparison to wild type controls ^⁎⁎⁎^p < .001 and Tukey comparison between groups ^#^p < .05, ^##^p < .01, ^###^p < .001. (For interpretation of the references to colour in this figure legend, the reader is referred to the web version of this article.)

Having previously demonstrated pan-neuronal expression of CHMP2B^Intron5^ resulted in a synaptic overgrowth phenotype at the *Drosophila* third instar larval NMJ, we employed this model to perform a small scale, high throughput, in vivo screen for compounds that alter CHMP2B^Intron5^ toxicity (Ahmad et al., 2009; Lee et al., 2007; Lu et al., 2013; West et al., 2015; West et al., 2018). As observed previously, pan-neuronal expression of CHMP2B^Intron5^ resulted in a significant increase in both synaptic bouton number and total synaptic arbour length in vehicle treated animals (Fig. 1A–E). Larvae expressing CHMP2B^Intron5^ also showed a significant increase in the number of branches emanating from the primary NMJ branch (Fig. 1A, F–G). Both UDCA and an analog compound Ursocholanic Acid (UCA) (Mortiboys et al., 2013), supplemented into the *Drosophila* food, exhibited a dose dependent rescue of all synaptic overgrowth phenotypes, compared to vehicle treated groups (Fig. 1A–G). A complete rescue of all aspects of synaptic overgrowth was achieved at a dose of 300 μM and 600 μM for UDCA and UCA, respectively (Fig. 1). Neither UDCA or UCA had any effect upon NMJ structure in wild type animals, with no significant variance in bouton number, branch number or NMJ length observed across doses (Fig. 1).

Having observed a dose dependent rescue of synaptic overgrowth at the *Drosophila* larval NMJ, we asked whether administration of UDCA or UCA was sufficient to alleviate impaired locomotor velocity observed previously in CHMP2B^Intron5^ expressing larvae (West et al., 2018). Administration of 600 μM of either UDCA or UCA was sufficient to partially rescue impaired crawling velocity in larvae pan-neuronally expressing CHMP2B^Intron5^ (Fig. 1H, I). Neither compound had a significant effect upon wild type larvae crawling behaviour. Further analysis revealed pan-neuronal expression of CHMP2B^Intron5^ resulted in a perturbation to normal crawling behaviour with larvae showing a significant increase in the number of distinct directional changes made during crawling (Fig. 1H&J). Both UDCA and UCA were sufficient to alleviate this phenotype, without perturbing normal crawling behaviour in wild type animals (Fig. 1H&J).

### 2.2 UDCA and UCA rescue dendritic collapse and cell death in mammalian CHMP2B^Intron5^ expressing neurons

**Fig. 2:**
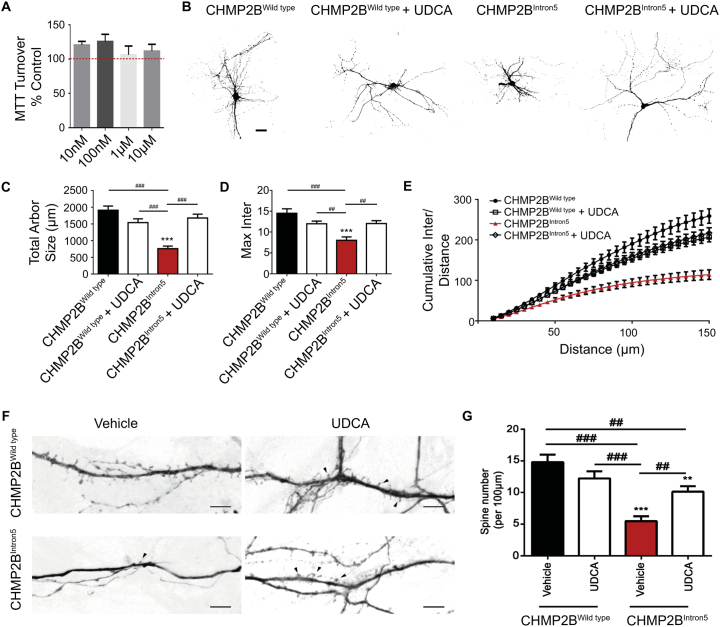
UDCA Prevents CHMP2B^Intron5^ Induced Dendritic Loss. A. UDCA does not cause mitochondrial toxicity (up to 10 μM, 48 h) as assessed by MTT turnover. Each treatment expressed as percentage of ethanol treated controls and shown as means ± SEM (*n* = 3 biological replicates). B. Representative micrographs of mature neurons expressing FLAG-tagged CHMP2B^Wildtype^ or CHMP2B^Intron5^ ± UDCA (10 μM, 48 h). Scale bar = 50 μm. C–E. Quantification of total arbour size (C) total number of intersections (D) and cumulative number of intersections per unit distance (E) in CHMP2B^Wildtype^ or CHMP2B^Intron5^ ± UDCA (10 μM, 48 h). Data represents mean ± SEM analysed using one-way ANOVA and Tukey's multiple comparisons post hoc test (^##^p < .01, ^###^p < .001). 30 neurons analysed, per condition, across 3 biological replicates. F. Representative micrographs of dendrites and dendritic spines expressing FLAG-tagged CHMP2B^Wildtype^ or CHMP2B^Intron5^ ± UDCA (10 μM, 48 h). Black arrowheads indicate dendritic spines. Scale bar = 5 μm. G. Quantification of dendritic spines per 100 μm. Data represents mean ± SEM analysed using one-way ANOVA and Tukey's multiple comparisons post hoc test (^##^p < .01, ^###^p < .001). Scale bar = 50 μm. 30 neurons analysed, per condition, across 3 biological replicates.

Having observed a dose dependent rescue of neuronal aberrations in *Drosophila* expressing the *CHMP2B*^*Intron5*^ mutant transgene, we asked whether similar effects could be recapitulated in mammalian models. Previously we have shown that CHMP2B^Intron5^ expression in primary rat neurons induces marked dendritic collapse and eventual apoptosis (Lee et al., 2007; West et al., 2018). In order to monitor the effect of UDCA on these neurons, we first assayed UDCA toxicity in primary neurons using MTT (3-(4,5-dimethylthiazol-2-yl)-2,5-diphenyltetrazolium bromide) assays. Primary neurons tolerated concentrations up to 10 μM (48 h) with no significant differences in MTT absorbance between groups (Fig. 2A). Next, we transfected rat neurons with FLAG-tagged CHMP2B^Wildtype^ or CHMP2B^Intron5^ and treated neurons with 10 μM UDCA or 0.1% ethanol (vehicle control). CHMP2B^Intron5^ expression decreased the complexity of the dendritic arbour, causing a significant decrease in the total size of the neuron (Fig. 2B,C). No significant differences were observed between CHMP2B^Wildtype^expressing cells treated with vehicle or UDCA. To understand whether CHMP2B^Intron5^expression affects dendritic branching, we assayed the maximum number of dendrites of each neuron (Fig. 2D, maximum intersections) and the cumulative number of branches per unit distance from the cell body, using Sholl analysis (Sholl, 1953). CHMP2B^Intron5^ transfected neurons showed a significant reduction in the maximum and cumulative number of intersections at any given distance from the cell body. Addition of 10 μM UDCA to the media post transfection was sufficient to alleviate these CHMP2B^Intron5^ associated phenotypes (Fig. 2D+E). To understand whether CHMP2B^Intron5^ expression induces changes in dendritic complexity that may alter the functional connectivity of neurons, we analysed the density of dendritic spines. CHMP2B^Intron5^ expression significantly reduced the number of spines per 100 μm of dendritic arbour (Fig. 2F). Treatment of neurons with 10 μM UDCA reversed the loss of dendritic spines (Fig. 2F+G). Using the chemically related compound UCA, we found a significant decrease in MTT turnover at 10 μM in primary neurons, however lower concentrations were well tolerated (Fig. S1A). CHMP2B^Intron5^ induced dendrite loss was also rescued using UCA (Fig. S1B, 1 μM, 48 h), as quantified by monitoring total arbour size, maximum intersections and cumulative intersections over distance (Fig. S1C–E).

While both UDCA and UCA exhibit neuroprotection in *Drosophila* and mammalian models of *CHMP2B*^*Intron5*^ related FTD, UDCA has FDA approval and is currently in clinical trial for both ALS and Parkinson's Disease. As such it represents a promising target for drug-repurposing for FTD and hereon we therefore focus upon UDCA.

### 2.3 UDCA ameliorates neuronal cell death

**Fig. 3:**
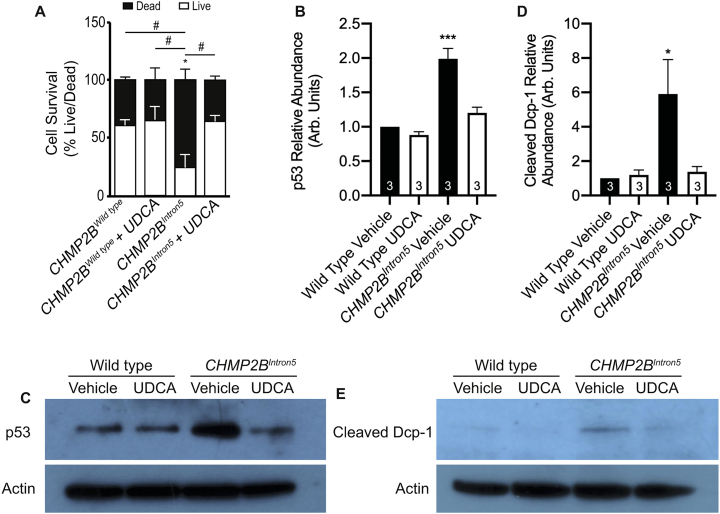
UDCA Alleviates Markers of Cell Death in CHMP2B^Intron5^ Models. A. Effect of CHMP2B^Wildtype^ or CHMP2B^Intron5^ ± UDCA (10 μM, 48 h) on the survival of mature cortical neurons. Data represents mean ± SEM analysed using two-way ANOVA followed by a Dunnett's (^⁎^p < .050) and Tukey's multiple comparisons post hoc test (^#^p < .05). 125 neurons analysed over three independent experiments. B—C. Relative p53 abundance in the Drosophila CNS of third instar larvae (Wildtype vs CHMP2B^Intron5^) raised on food supplemented with either vehicle or UDCA (600 μM). B. Quantification of 3 independent experiments blotting p53 from Drosophila lysates. Normalised against actin loading control and relative to vehicle treated wild types. ANOVA with post-hoc Dunnett's comparison to wild type controls ^⁎⁎⁎^p < .001. C. Representative immunoblot of p53. D, E. Relative Cleaved Death Caspase 1 (Dcp-1) abundance in the Drosophila CNS of third instar larvae (Wild type vs CHMP2B^Intron5^) raised on food supplemented with either vehicle or UDCA (600 μM). D. Quantification of 3 independent experiments blotting cleaved Dcp-1 from *Drosophila* lysates. Normalised against actin loading control and relative to vehicle treated wild types. ANOVA with post-hoc Dunnett's comparison to wild type controls ^⁎^p < .05. E. Representative immunoblot of cleaved Dcp-1.

**Supplementary Fig. 2:**
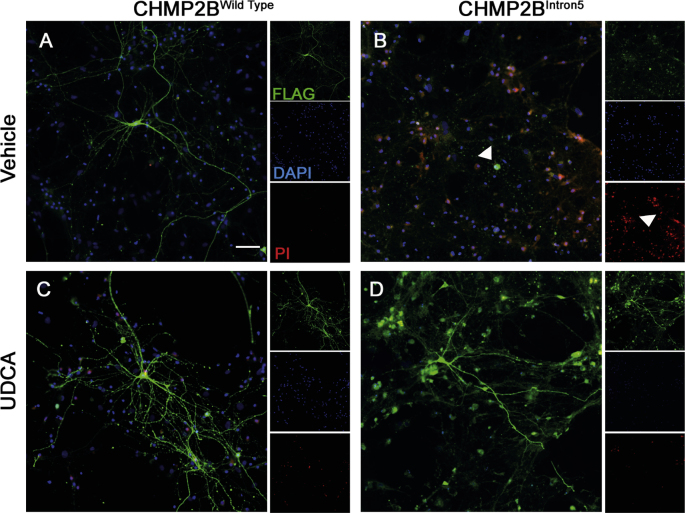
UCA Prevents CHMP2B^Intron5^ Induced Cell Death. A-D Representative micrographs of neurons expressing FLAG-tagged CHMP2B^Wildtype^ or CHMP2B^Intron5^ (FLAG, Green) ± UDCA (10 μM, 48 h) stained with propidium iodide (PI - red). Nuclei counterstained with DAPI (blue). Scale bar = 50 μm.

Having observed the protective effects of UDCA at the behavioural, dendritic and synaptic level, we asked whether UDCA could ameliorate cell death induced by CHMP2B^Intron5^ expression. Previous studies have demonstrated expression of CHMP2B^Intron5^ in primary neurons severely affects neuronal survival (Lee et al., 2007). Using propidium iodide exclusion assays, we found that CHMP2B^Intron5^ expression induced a similar level of cell death, with 75% of nuclei in CHMP2B^Intron5^ expressing neurons staining positive for propidium iodide. Addition of UDCA significantly decreased the number of dead cells, bringing survival back to wild type levels (Fig. 3A, Supplementary Fig. 2).

Perturbed regulation of apoptosis has been proposed as a mechanism driving neuronal cell loss in FTD and ALS. Previously we identified elevated apoptotic cascades in both *Drosophila* and mammalian neurons expressing CHMP2B^Intron5^ (West et al., 2018). UDCA has been proposed to act as a potent anti-apoptotic agent, acting to regulate p53 levels (Amaral et al., 2010). Having shown UDCA can alleviate CHMP2B^Intron5^ dependent cell death in primary neurons (Fig. 3A) we asked whether UDCA could ameliorate expression of apoptotic markers, including p53 accumulation and cleavage of the *Drosophila* caspase 3 homologue, Death Caspase-1 (Dcp-1) (Fig. 3B–E). Pan-neuronal expression of CHMP2B^Intron5^ led to a significant increase in both p53 and cleaved Dcp-1 in the *Drosophila* larval CNS, both of which were rescued by UDCA administration (Fig. 3B–E).

### 2.4 UDCA does not alleviate CHMP2B^Intron5^ induced accumulation of ubiquitin positive inclusions or autophagosomes

**Fig. 4:**
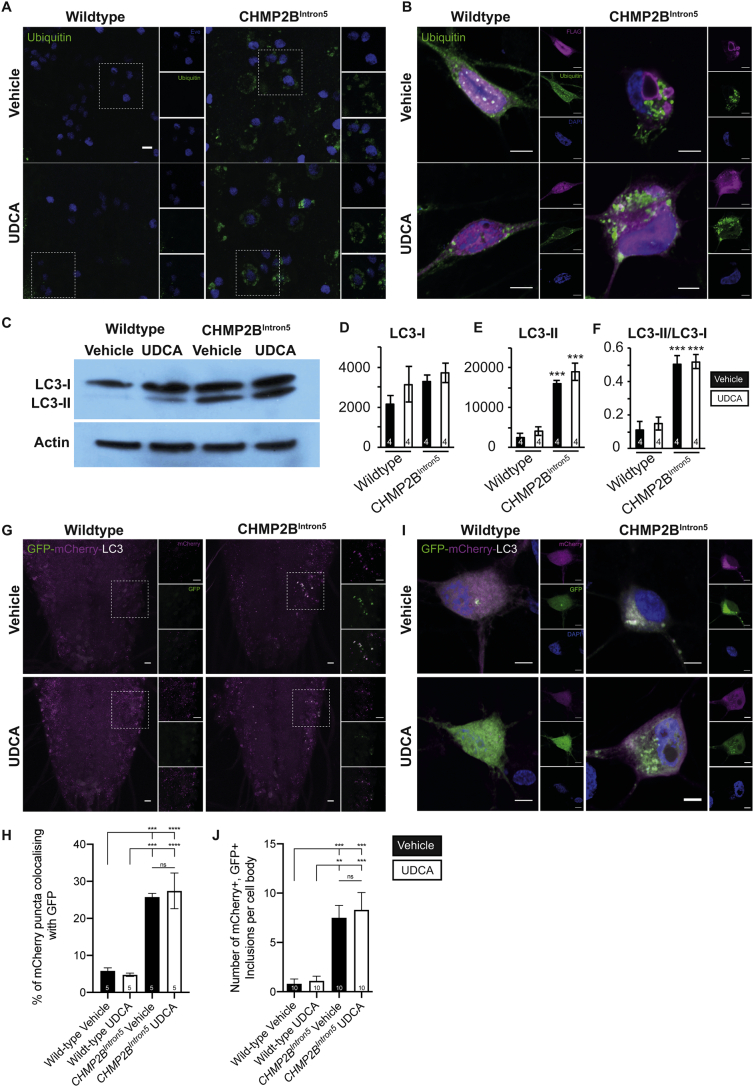
UDCA Does Not Rescue Accumulation of Ubiquitin-positive Aggregates and Autophagosomes In Drosophila and Mammalian Neurons Expressing CHMP2B^Intron5^. A. Ubiquitinated proteins in the CNS of wild type and CHMP2B^Intron5^ expressing (*nSyb*-Gal4) *Drosophila* third instar larvae raised on vehicle (ethanol) or UDCA (600 μM) supplemented food. *Drosophila* motor neurons are labelled using an *eve*-eGFP endogenous reporter (blue). Scale bars = 5 μm. B. Representative micrographs of mature neurons expressing FLAG-tagged CHMP2B^Wildtype^ or CHMP2B^Intron5^ (FLAG, magenta) ± UDCA (10 μM, 48 h) stained for ubiquitinated (green) proteins. Nuclei counterstained with DAPI (blue). Scale bar = 5 μm. C—F. Quantification of LC3-I and LC3-II in the Drosophila larval CNS. Immunoblots (C) from 4 independent biological replicates were quantified for cytosolic LC3-I (D) and LC3-phosphatidylethanolamine conjugate (LC3-II) (E), which is recruited to autophagosomal membranes. F. LC3-I/LC3-II ratio. *n* = 4. All Normalised against actin loading. ANOVA with post-hoc Dunnett's comparison to wild type controls ^⁎⁎⁎^p < .001, ^⁎⁎⁎⁎^p < .0001. G, H. Autophagic flux in the CNS of wild type and CHMP2B^Intron5^ expressing (*nSyb*-Gal4) *Drosophila* third instar larvae raised on vehicle (ethanol) or UDCA (600 μM) supplemented food. The dual tagged GFP-mCherry-LC3/Atg8a labels autophagosomes (GFP and mCherry), amphisomes (mCherry only) and autolysosomes (mCherry only). Scale bars = 10 μm. Quantification (H) of the percentage of LC3 aggregates positive for both mCherry and GFP in the Drosophila larval nervous system. ANOVA with post-hoc Tukey's comparison between groups ^⁎⁎⁎^p < .001, ^⁎⁎⁎⁎^p < .0001. Quantification from 5 independent animals per condition (*N* = 5). I. Representative micrographs of mature neurons expressing FLAG-tagged CHMP2B^Wildtype^ or CHMP2B^Intron5^ (FLAG, magenta), co-transfected with a plasmid encoding GFP-mCherry-LC3. Cells were treated with vehicle (0.1% ethanol) or UDCA (10 μM, 48 h) and stained for GFP (green) and mCherry (red). Autophagosome accumulation was monitored by observing the presence of green and red puncta. Nuclei counterstained with DAPI (blue). Scale bar = 5 μm. J. Quantification of micrographs in (I) showing the number of GFP and mCherry double positive aggregates per cell. *n* = 10 cells per condition. ANOVA with post-hoc Tukey's comparison between groups ^⁎⁎⁎^p < .001, ^⁎⁎^p < .01. (For interpretation of the references to colour in this figure legend, the reader is referred to the web version of this article.)

Having shown UDCA ameliorates synaptic overgrowth, dendritic collapse, neuronal cell death and apoptotic-cascades, we asked whether it modified two pathological hallmarks of CHMP2B^Intron5^ toxicity, the aberrant accumulation of autophagic organelles and ubiquitin positive inclusions within the nervous system (Holm et al., 2007; Lee et al., 2007). Accumulation of ubiquitinated inclusions was observed within neurons in the *Drosophila* larval nervous system (Fig. 4A) and in mammalian primary neurons expressing CHMP2B^Intron5^ (Fig. 4B). UDCA showed no ability to alleviate the accumulation of ubiquitinated inclusions in either model (Fig. 4A, B).

Previous studies have suggested that the therapeutic action of UDCA in the treatment of hepatic fibrosis may be through inhibition of autophagy (Ye et al., 2020). We therefore looked to ascertain whether the neuroprotective mode of action of UDCA in *CHMP2B*^*Intron5*^ models worked via the modulation of autophagy and could alleviate aberrant autophagosome accumulation, a hallmark of *CHMP2B*^*Intron5*^ FTD. In order to quantify CHMP2B^Intron5^-dependent perturbations to autophagy and the effects of UDCA treatment, we quantified the abundance of the cytosolic and autophagosomal forms of LC3 in the Drosophila nervous system. LC3 (LC3-I) is conjugated to phosphatidylethanolamine (PE) in order to recruit LC3 to autophagosomal membranes. Fusion of autophagosomes with lysosomes results in degradation of the PE-conjugated form (LC3-II). Due to the altered hydrophobicity of the conjugated form, LC3-II is identifiable as a distinct band from LC3-I via immunoblotting. In larvae pan-neuronally expressing CHMP2B^Intron5^ we observed a significant increase in the amount of LC3-II present in the nervous system, compared to wildtype (Fig. 4C–F) which was not alleviated by UDCA treatment. Having demonstrated accumulation of autophagic material in CHMP2B^Intron5^expressing flies we used the pH sensitive tandem tagged GFP-mCherry-LC3 dual colour system as a reliable system with which to investigate autophagic flux (Nezis et al., 2010). This construct works on the principle that fluorescence from the pH-sensitive GFP tag to LC3/Atg8 will be quenched upon autophagosome fusion with lysosomes, while the pH insensitive mCherry tag will not. Thus an increase in the number of GFP-mCherry double labelled puncta suggests an impairment to autophagic flux (Klionsky et al., 2016; Nezis et al., 2010; Pankiv et al., 2007). *Drosophila* larvae pan-neuronally expressing CHMP2B^Intron5^ showed an aberrant accumulation of large GFP-mCherry double labelled puncta within the ventral nerve cord (VNC)(Fig. 4G, H). In contrast, GFP-positive puncta were rarely observed in wild type animals. Administration of UDCA had no significant effect upon the number of GFP-mCherry labelled puncta observed in CHMP2B^Intron5^ expressing larvae, compared to vehicle treated controls (4G-H). Similarly in rat primary hippocampal neurons, co-transfection of CHMP2B^Intron5^ with GFP-mCherry-LC3 produced large GFP-mCherry positive puncta, which were not affected by administration of 10 μM UDCA (Fig. 4I, J). CHMP2B^Wildtype^expressing controls showed diffuse GFP and mCherry fluorescence, with few visible aggregates or puncta observed.

**Fig. 5:**
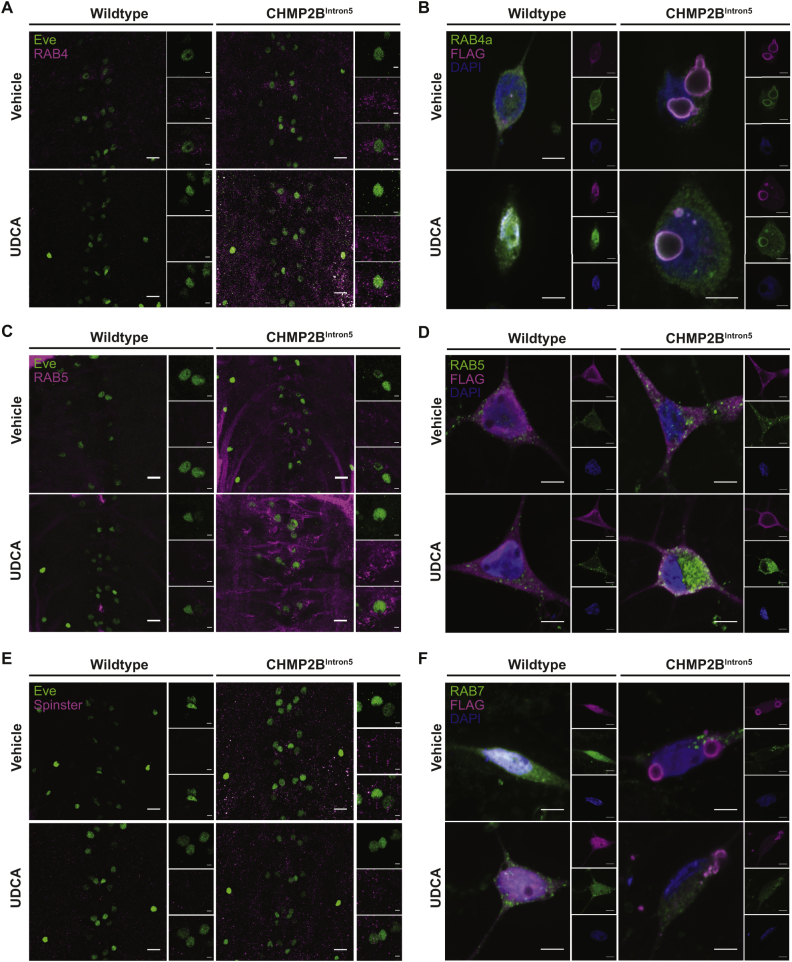
UDCA Does Not Rescue Endosomal Perturbations in neurons expressing CHMP2B^Intron5^. A. Representative micrographs of RAB4 positive endosomes (early/recycling) in the CNS of Wild type and CHMP2B^Intron5^ expressing (*nSyb*-Gal4) larvae raised on vehicle (ethanol) or UDCA (600 μM) supplemented food. Scale bars: main panel = 10 μm, inset = 2 μm. B. Representative micrographs of mature neurons expressing FLAG-tagged CHMP2B^Wildtype^ or CHMP2B^Intron5^ (FLAG, magenta) ± UDCA (10 μM, 48 h) stained for RAB4a (early/recycling endosome, green) protein. Nuclei counterstained with DAPI (blue). Scale bar = 5 μm. C. Representative micrographs of RAB5 positive endosomes (early) in the CNS of Wild type and CHMP2B^Intron5^ expressing (*nSyb*-Gal4) larvae raised on vehicle (ethanol) or UDCA (600 μM) supplemented food. Scale bars: main panel = 10 μm, inset = 2 μm. D. Representative micrographs of mature neurons expressing FLAG-tagged CHMP2B^Wildtype^ or CHMP2B^Intron5^ (FLAG, magenta) ± UDCA (10 μM, 48 h) stained for RAB5 (early endosome, green) protein. Nuclei counterstained with DAPI (blue). Scale bar = 5 μm. E. Representative micrographs of the late endosome/lysosomal marker Spinster in the CNS of Wild type and CHMP2B^Intron5^ expressing (*nSyb*-Gal4) larvae raised on vehicle (ethanol) or UDCA (600 μM) supplemented food. Scale bars: main panel = 10 μm, inset = 2 μm. F. Representative micrographs of mature neurons expressing FLAG-tagged CHMP2B^Wildtype^ or CHMP2B^Intron5^ (FLAG, magenta) ± UDCA (10 μM, 48 h) stained for RAB7 (late endosome, green) proteins. Nuclei counterstained with DAPI (blue). Scale bar = 5 μm. (For interpretation of the references to colour in this figure legend, the reader is referred to the web version of this article.)

### 2.5 UDCA acts downstream of endosomal dysfunction

**Supplementary Fig. 3:**
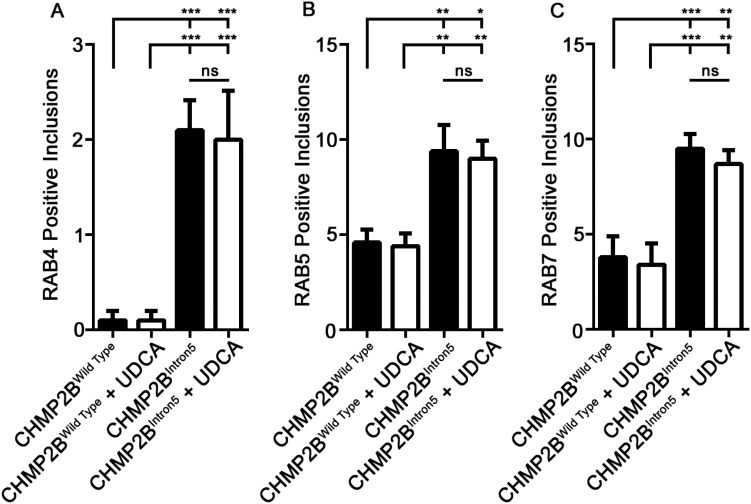
UDCA does not Rescue Endosomal Perturbations in Primary Neurons. Quantification of RAB4 (A) RAB5 (B) and RAB7 (C) positive inclusions in neurons transfected with FLAG-tagged CHMP2B^Wildtype^ or CHMP2B^Intron5^ ± UDCA (10 μM, 48 h). Data represents mean ± SEM analysed using one-way ANOVA and Tukey's multiple comparisons post hoc test (^⁎^p < .05, ^⁎⁎^p < .01, ^⁎⁎⁎^p < .001). 10 neurons analysed, per condition, across 3 biological replicates.

The C-terminal truncation of CHMP2B^Intron5^ prevents both the assembly and disassembly of ESCRT complexes. As such, expression of CHMP2B^Intron5^ has been shown to cause the accumulation of polyubiquitinated proteins that co-localise with the late endosomal marker, RAB7 (Lee and Gao, 2009). These RAB7 positive endosomes are enlarged in CHMP2B^Intron5^ patient fibroblasts and fail to fuse with the lysosome (Urwin et al., 2010). Similarly, CHMP2B^Intron5^ expression in *Drosophila* induces swelling of RAB5 and RAB7 positive endosomes (Lee and Gao, 2009). To determine if UDCA could prevent CHMP2B^Intron5^ dependent endosomal aberrations we labelled endosomes in both wild type and CHMP2B^Intron5^ expressing *Drosophila* and mammalian neurons treated with vehicle and UDCA. VNC's of wild type or *CHMP2B*^*Intron5*^ larvae were stained with antibodies for RAB4 (early/recycling endosomes (Schmidt and Haucke, 2007)), RAB5 (early endosomes) and Spinster (a late endosomal/lysosomal marker (Sweeney and Davis, 2002)). Mammalian neurons were labelled with RAB4a (early/recycling endosomes), RAB5 (early endosomes) and RAB7 (late endosomes). In larval VNC's, CHMP2B^Intron5^ induced aggregates of RAB4 (Fig. 5A). Similarly, in mammalian neurons, CHMP2B^Intron5^ induced large vacuole type structures, as previously reported (Urwin et al., 2010), that co-localise with RAB4a. The clustering of RAB4 in *Drosophila* and mammalian CHMP2B^Intron5^ neurons was unchanged by the addition of UDCA (Fig. 5A+B, Supplementary Fig. 3A). In both *Drosophila* and mammalian neurons, CHMP2B^Intron5^ induced large aggregates of RAB5 positive endosomes, which were unaffected by UDCA (Fig. 5C+D, Supplementary Fig. 3B). Similarly, in *Drosophila*, CHMP2B^Intron5^ induced *Spin* positive aggregates throughout the VNC that were not obviously rescued by UDCA (Fig. 5E). In mammalian neurons, CHMP2B^Intron5^ formed large intracellular vacuoles that partially colocalized with aggregates of RAB7. Enlarged RAB7 positive endosomes were not changed by the addition of UDCA (Fig. 5E+F, Supplementary Fig. 3C).

## 3 UDCA identifies GCLC as a novel regulator of CHMP2B^Intron5^ toxicity

**Fig. 6:**
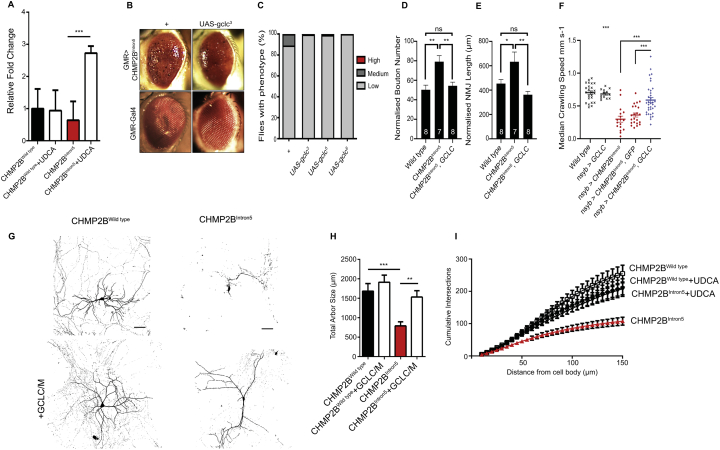
UDCA Identifies GCLC As a Novel Regulator of CHMP2B^Intron5^ Toxicity. A. The mRNA abundance of *gclc* from pan-neuronally expressing CHMP2B^Intron5^ third instar larval brains shows a significant upregulation when raised on UDCA (600 μM) supplemented food compared to vehicle (ethanol). Mean ± SEM, *n* = 5 per genotype, fold change values in log_2_ scale. ^⁎⁎⁎^ q < 0.001. B. Representative images of the *Drosophila* eye phenotype caused by CHMP2B^Intron5^ expression with eye-specific *GMR*-Gal4 driver (*GMR*-Gal4, UAS-CHMP2B^Intron5^) and amelioration by co-expression of UAS-*GCLC*. C. Quantification of the eye phenotype from (B) genotypes. *n* = 100. D, E. Co-expression of GCLC (UAS-*GCLC*^6^) ameliorates unregulated synaptic growth characterised by increased bouton number (D) and NMJ length (E) at the third instar larval NMJ (Muscle 6/7, hemisegment A3) in CHMP2B^Intron5^ (*nSyb*-Gal4) expressing larvae. ANOVA with post-hoc Tukey comparison between groups ^⁎^p < .05, ^⁎⁎^p < .01. F. Pan-neuronal (*nSyb*-Gal4) expression of GCLC rescues aberrant crawling behaviour in 3rd instar wandering larvae pan-neuronally expressing CHMP2B^Intron5^. ANOVA with post-hoc Dunnett's comparison to wild type controls ^⁎⁎⁎^p < .001 and Tukey comparison between groups ^###^p < .001. G. Representative micrographs of mature neurons expressing FLAG-tagged CHMP2B^Wildtype^ or CHMP2B^Intron5^ ± plasmids expressing the catalytic (GCLC) and modifying (GCLM) subunits of glutamate cysteine ligase (GCL). H,  I Quantification of total arbour size (H) and cumulative number of intersections per unit distance (I) in CHMP2B^Wildtype^ or CHMP2B^Intron5^ ± GCLC/M. Data represents mean ± SEM analysed using one-way ANOVA and Tukey's multiple comparisons post hoc test (^##^p < .01, ^###^p < .001). Scale bar = 50 μm. 20 neurons analysed, per condition, across 3 biological replicates.

**Supplementary Fig. 4:**
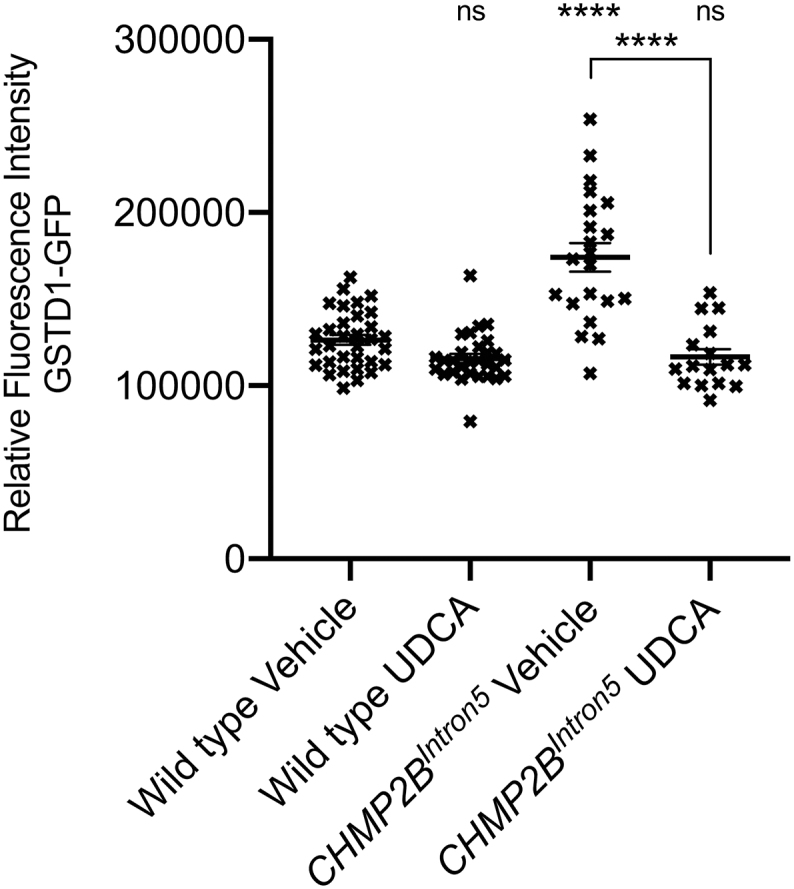
UDCA Rescues Increased *Glutathione S-TransferaseD1-*GFP Reporter Fluorescence Intensity in CHMP2B^Intron5^ Expressing Larvae. Quantification of *GstD1*-GFP reporter expression in wild type and *nSyb* > CHMP2B^Intron5^ third instar larvae fed either vehicle or UDCA. ANOVA with Tukey's multiple comparisons post hoc test (^⁎⁎⁎⁎^p < .0001).

Having observed little effect of UDCA upon dysfunctional endosomal-lysosomal and autophagic pathways in CHMP2B^Intron5^ expressing *Drosophila* and mammalian neurons, we looked to determine downstream pathways in which UDCA may act to alleviate the neuronal phenotypes observed in our models. RNA-sequencing (RNA-seq) of VNC's dissected from larvae pan-neuronally expressing wild type or CHMP2B^Intron5^, raised on UDCA or vehicle supplemented food was carried out. The main target identified using RNA sequencing was the catalytic subunit of glutamate cysteine ligase (GCLC) which significantly increased in CHMP2B^Intron5^ expressing larvae fed UDCA in comparison to vehicle controls (Fig. 6A). Additional RNA sequencing data is available in supplementary Table 1. Having observed an increase in *GCLC* gene expression in CHMP2B^Intron5^ expressing larvae raised on UDCA supplemented food, a dominant modifier screen, using our established *Drosophila* eye expression model (Ahmad et al., 2009), was used to ask whether there was a functional genetic interaction between GCLC and CHMP2B^Intron5^ expression. CHMP2B^Intron5^ dependent degeneration of the fly eye was suppressed by co-expression of three independent UAS-*GCLC* (Orr et al., 2005) transgenes (Fig. 6B+C). Having established a genetic interaction between CHMP2B^Intron5^ expression and GCLC, we asked whether GCLC exhibited a functional role in the unregulated neuronal growth phenotypes observed in larvae pan-neuronally expressing CHMP2B^Intron5^. Co-expression of GCLC was sufficient to alleviate all aspects of synaptic overgrowth at the *Drosophila* larval NMJ, including increased NMJ length and bouton number (Fig. 6D+E). Pan-neuronal expression of GCLC was also sufficient to rescue median crawling speed to near wild type control levels (Fig. 6F). Elevated levels of the oxidative stress responsive Glutathione S-Transferase reporter GstD1-GFP also observed in CHMP2B^Intron5^ expressing larvae, and this increased expression was also rescued by UDCA administration (Supplementary Fig. 4). Having characterised GCLC as a positive regulator of CHMP2B^Intron5^ toxicity in *Drosophila*, we asked whether overexpression of GCLC in primary mammalian neurons could rescue dendritic collapse phenotypes associated with the mutation. We found that co-expression of both the catalytic and modifying subunits of GCL (GCLC and GCLM) with CHMP2B^Intron5^ was sufficient to rescue dendrite loss (Fig. 6G), as analysed by measuring total arbor size (Fig. 6H) and total number of intersections per unit distance (Fig. 6I).

## 4 Discussion

Defective endosomal-lysosomal trafficking is implicated in numerous neurodegenerative diseases, including FTD and ALS (Lee et al., 2013; Nixon, 2005). Of particular importance are late endosomes, where MVBs regulate transmembrane protein sorting and exosome release. MVB formation is controlled by the ESCRT complexes, in which CHMP2B plays a critical role (Babst, 2011; Hurley and Emr, 2006; Lee and Gao, 2008). This process, where ubiquitinated vesicle cargoes are internalised and degraded via the lysosome represents an evolutionarily highly conserved biological process in eukaryotic cells (Leung et al., 2008). As such, mutations that affect core components of the ESCRT machinery are either lethal (CHMP4/6) (Schmidt and Teis, 2012), or involve severe accumulation of proteins which eventually leads to defective endosomal trafficking, autophagy and cell death (CHMP2B). The importance of ESCRT integrity can be observed when considering aggregated protein hallmarks across the FTD-ALS spectrum. Up to 97% of sporadic ALS patients display neuronal inclusions of polyubiquitinated TDP-43, a major driver of ALS pathology (Prasad et al., 2019). Functional ESCRT subunits and MVB formation is required for the clearance of TDP-43 (Filimonenko et al., 2007). While *CHMP2B* mutations are rare, dysfunctional endosomal-lysosomal signalling is common across the FTD-ALS spectrum, pointing to an essential role for ESCRT-III function in the maintenance of neuronal health. Therefore, identifying therapeutics that prevent or delay neurodegeneration associated with CHMP2B^Intron5^ mutations have potential across the FTD-ALS spectrum. UDCA has been identified as a potential therapeutic compound for drug-repurposing for the treatment of ALS, Parkinson's disease and Alzheimer's Disease. However, despite the initiation of clinical trials, the mode of action of UDCA remains unclear. Here we reveal UDCA conveys neuroprotection in *Drosophila* and mammalian models of *CHMP2B*^*Intron5*^ FTD, identifying novel aspects of CHMP2B^Intron5^ pathology and identifying neuroprotective pathways mediated by UDCA. The observation that UDCA protects neurons without rectifying endosomal-lysosomal and autophagic dysfunction also highlights its potential as a therapeutic downstream of compromised proteostasis, a common feature of many neurodegenerative conditions.

### 4.1 UDCA rescues behavioural, synaptic and dendritic aberrations induced by CHMP2B^Intron5^

UDCA is a natural hydrophilic bile acid produced in the gut. It is also an FDA-approved drug primarily used in the treatment of primary biliary cholangitis. However, it has also been shown to be protective in cell and animal models of AD and PD (Bell et al., 2018; Graham et al., 2018; Lo et al., 2013; Ramalho et al., 2006). Here we demonstrate that UDCA acts as a neuroprotectant against neuronal perturbations and cell death in both *Drosophila* and mammalian models of FTD caused by *CHMP2B*^*Intron5*^. mutations.Administration of UDCA to *Drosophila* pan-neuronally expressing CHMP2B^Intron5^, exhibits a dose dependent rescue of NMJ overgrowth and locomotor dysfunction. Furthermore, UDCA prevents dendritic retraction and spine loss in mammalian primary neurons expressing CHMP2B^Intron5^.

Neuronal apoptosis has been identified in *CHMP2B*^*Intron5*^ related FTD (West et al., 2018). UDCA exhibits anti-apoptotic properties including p53 modulation, independent of cell type (Amaral et al., 2009). We report here that UDCA prevents elevated p53 and cleaved Dcp-1 expression in CHMP2B^Intron5^expressing larvae and partially rescues neuronal death induced by CHMP2B^Intron5^expression in mammalian neurons. Neuronal inclusions are a hallmark of neurodegenerative diseases, associated with perturbed proteostasis and dysfunctional endosomal-lysosomal and autophagic pathways. Dendrite retraction and autophagosome accumulation has been reported in mature neurons (10-15DIV) expressing CHMP2B^Intron5^ (Lee et al., 2007; Belly et al., 2010; West et al., 2018) while younger neurons (7DIV) show a marginal increase in dendritic branches when expressing CHMP2B^Intron5^ (Clayton et al., 2018). Treating our 15DIV neurons expressing CHMP2B^Intron5^ with UDCA or UCA rescues dendritic retraction. We show that in our *CHMP2B*^*Intron5*^ models of FTD, autophagosome accumulation precedes ubiquitin positive inclusions in neurons (Ghazi-Noori et al., 2012; Lee et al., 2007). To evaluate if UDCA ameliorated neuronal cell death and apoptotic-cascades by promoting endosomal-lysosomal trafficking and/or autophagic flux, we examined the effect of UDCA by monitoring endosomal and autophagic markers in both *Drosophila* and mammalian primary neurons expressing CHMP2B^Intron5^.

### 4.2 UDCA acts downstream of a dysfunctional endosomal-lysosomal and autophagic system in *CHMP2B*^*Intron5*^ models

UDCA has been shown to both promote (Lim and Han, 2015; Pang et al., 2017; Panzitt et al., 2020; Wang et al., 2017) and inhibit autophagic flux (Ye et al., 2020). The *CHMP2B*^*Intron5*^ mutation results in significant perturbations to endosomal-lysosomal (Urwin et al., 2010) and autophagosomal trafficking (Filimonenko et al., 2007; Ghazi-Noori et al., 2012; Lee et al., 2007). However, administration of UDCA showed no significant effect upon autophagic flux in *Drosophila* or mammalian primary neuronal models of *CHMP2B*^*Intron5*^ related FTD. Furthermore, UDCA failed to alleviate aberrant accumulation of endosomes and ubiquitinated protein inclusions, a hallmark of perturbed proteostasis in neurodegenerative diseases. Despite this, UDCA was sufficient to alleviate synaptic overgrowth, dendritic retraction, cell death and behaviour deficits in these models. These findings support the hypothesis that UDCA acts downstream of endosomal-lysosomal and autophagic dysfunction, making it an attractive target for drug-repurposing as a general neuroprotectant. One consideration in regards to the discrepancy between this study and previous studies looking at the effect of UDCA upon autophagic flux is that the majority of experiments looking at the role of UDCA on autophagy have been performed in hepatic or cancer cell lines (Panzitt et al., 2020). It is important to consider that the metabolic energy demand and source of energy in neurons is distinct from other cell types, with neurons in the CNS less dependent upon autophagy as a source of amino acids and energy (Boland and Nixon, 2006; Yue et al., 2009). Furthermore, there exists complex interplay between autophagy and the cell cycle in mitotic cells that may be absent in neurons (Mathiassen et al., 2017). As a result, the regulation of autophagic flux in neurons and non-neuronal cells is likely to depend upon distinct molecular mechanisms and cannot be directly compared (Yue et al., 2009). Having shown no effect upon autophagy in two independent models of CHMP2B-related FTD we propose the ability of UDCA to rescue neuronal aberrations in these models is independent and downstream of autophagy. Furthermore we show UDCA has no effect upon endosomal perturbations and aberrant accumulation of ubiquitin-positive inclusions, supporting the hypothesis that UDCA acts downstream of perturbed endosomal-lysosomal and autophagic dynamics associated with *CHMP2B*^*Intron5*^ disease causing mutations. While our data supports this hypothesis, it is important to note that UDCA may also act via an undefined parallel pathway to exert neuroprotection.

### 4.3 UDCA identifies GCLC as a novel regulator of CHMP2B^Intron5^ toxicity

Reactive oxygen species (ROS) are bi-products of cellular metabolism and play pivotal roles in physiology and pathology. In the CNS, ROS are neutralized by a myriad of reductive mechanisms that operate in neurons and glia, including the glutathione (GSH) system. Under physiological conditions, ROS have been demonstrated to support the growth and plasticity of neurons (Oswald et al., 2018), however in many neurodegenerative disorders the reductive capacity of neurons is overwhelmed leading to prolonged oxidative stress, which contributes to disease progression. GSH homeostasis is altered in many neurodegenerative diseases (Aoyama and Nakaki, 2013; Gu et al., 2015) and decreased GSH levels are notable in the hippocampus of Alzheimer's disease patients (Mandal et al., 2015) and in mouse models of AD (Resende et al., 2008).

Prolonged oxidative stress damages lipids and lipid oxidation is a hallmark of many neurodegenerative diseases, including FTD. Increased markers of oxidized lipids have been detected in the cortex of patients with FTD compared to age matched controls. This lipoxidative damage is a marker of impaired mitochondrial function (Martínez et al., 2008). Recently, lipidomics of blood serum from FTD patients identified mitochondrial dysfunction, inflammation and oxidative stress as key aspects of FTD pathophysiology (Phan et al., 2020). More specifically, accumulation of ROS and subsequent neuronal damage has been identified in *CHMP2B*^*Intron5*^ patient derived neurons (Zhang et al., 2017). These neurons contain aberrant mitochondria, with reduced respiratory capacity and an increase in oxidative stress compared to controls. These data implicate mitochondrial impairment and oxidative damage in the pathophysiology of FTD. Boosting neuronal antioxidant defences, particularly intracellular glutathione levels, presents an attractive therapeutic target for FTD. Glutamate cysteine ligase (GCL) is the rate limiting enzyme in the GSH biosynthetic pathway. It has been known for some time that increasing the catalytic (GCLC) and modifying (GCLM) levels globally (Orr et al., 2005) or specifically in neurons (Moskalev et al., 2019) extends longevity in *Drosophila* by between 25 and 50%. Overexpressing GCLM increases cellular glutathione content 2-fold, protecting cells from oxidative stress (Moskalev et al., 2019). GCLC deficiency causes motor neuron loss, spinal cord atrophy and defective gait. Conditional reduction of GCLC in the forebrain causes neuronal atrophy, deficits in nesting behaviour and mitochondrial dysfunction (Feng et al., 2017), indicating a critical function for glutathione levels and the activity of the rate limiting enzyme GCL in the maintenance of neuronal health.

UDCA has been implicated in many different signalling pathways and has been shown to prevent oxidative stress (Brito et al., 2008) and negatively regulates p53 assembly (Amaral et al., 2010). UDCA has also been shown to ameliorate mitochondrial dysfunction in Parkinson's (Mortiboys et al., 2015) and Alzheimer's patient fibroblasts (Bell et al., 2018). Although the exact mechanism of action remains unknown, UDCA in our hands positively regulated the expression of GCL in *CHMP2B*^*Intron5*^ models. Furthermore, increasing GCL in both *Drosophila* and mammalian neurons, protects neurons from cell death. While these data demonstrate the protective effect of UDCA in a model of FTD, we are unable to identify specifically where UDCA acts in neurons. Identifying the receptor which binds UDCA and its downstream signalling, would have clear therapeutic potential for neurodegenerative disease, including FTD.

## 5 Conclusion

The data presented here demonstrates the neuroprotective properties of UDCA in both *Drosophila* and mammalian *CHMP2B*^*Intron5*^ models of FTD. UDCA prevents dendritic aberrations and apoptosis downstream of the main endosomal-lysosomal and autophagic hallmarks of the mutation. UDCA induced neuroprotection in *CHMP2B*^*Intron5*^ FTD highlights GCL and glutathione homeostasis as a novel regulator of FTD pathology. Although the receptor mediating the effects of UDCA remains unknown, its ability to protect neurons downstream of defective proteostasis identifies it as an attractive therapeutic with relevance across the FTD-ALS spectrum.

## 6 Materials and methods

### 6.1 *Drosophila*

#### 6.1.1 Stocks and husbandry

*Drosophila* were raised on 4–24® instant *Drosophila* medium (Carolina Biological Supply Company, USA) supplemented (50% *v/v*) with a yeast sucrose solution (5% *w/v* inactivated yeast, 10% *w*/*v* sucrose in ddH_2_O) at 25 °C on a 12 h light:dark cycle. Prior to mixing with 4–24® instant media, but post autoclaving and cooling, vehicle (Ethanol), UCA (Sigma, C7628) or UDCA (Sigma, U5127) were added to the yeast sucrose solution at the desired concentration. The final concentration of Ethanol was 0.06%. UAS-*CHMP2B*^*Intron5*^ flies were described previously (Ahmad et al., 2009; West et al., 2015; West et al., 2018). GFP-mCherry-LC3/Atg8a flies were a kind gift from Dr. Ioannis Nezis (Warwick, UK) (Nezis et al., 2010). *Even-skipped*-eGFP flies (y^1^ w*; PBac{*eve*-EGFP·S}VK00033, RRID:BDSC_30871) were obtained from the Bloomington Drosophila Stock Center. UAS-*GCLC* flies (GCLC 6, 3 and 5) were a gift from Professor William C. Orr (Orr et al., 2005). *nSyb*-Gal4 and *GMR*-Gal4 driver lines were described previously (Ahmad et al., 2009; West et al., 2015; West et al., 2018).

#### 6.1.2 Immunohistochemistry and NMJ analysis

Third instar wandering larvae were dissected, fixed, antibody stained, imaged and analysed as described previously (West et al., 2015). All NMJ analysis was performed double-blind. Primary antibodies used were Cy3-Conjugated anti-HRP (Goat, 1:200, Jackson ImmunoResearch Labs Cat^#^ 123–165-021, RRID:AB_2338959), anti-synaptotagmin (Rabbit, 1:2000, Syt-91, RRID:AB_2713991, (West et al., 2015)) anti-polyubiquitinated proteins (Mouse, 1:2000, FK2, Enzo Life Sciences Cat^#^ BML-PW8810–0500, RRID:AB_2051891), anti-RAB4 (Rabbit 1:100, Abcam Cat^#^ ab78970, RRID:AB_2042753), anti-RAB5 (Rabbit, 1:500, Abcam Cat^#^ ab31261, RRID:AB_882240) and anti-Spinster (Guinea Pig, 1:1000, RRID:AB_2833057, (Sweeney and Davis, 2002)). *Drosophila* motor-neurons were labelled using GFP-tagged *even-skipped* (*eve*). Confocal microscopy was performed using a Zeiss LSM 880 on an Axio Observer.Z1 invert confocal microscope (Zeiss). *Z*-stacked projections of NMJs and VNCs were obtained using a Plan Neofluar 40×/0.75 NA oil objective. NMJ lengths were measured from stacked NMJ images using the NeuronJ plugin for ImageJ (National Institutes of Health) as described previously (West et al., 2015; West et al., 2018).

#### 6.1.3 Larval locomotor assay

Larval locomotor assays were performed as described previously (West et al., 2018). Two to three larvae were transferred onto the centre of a 90 mm diameter petri-dish containing a thin layer of 1% agar and left to acclimatize. The petri dish was placed upon a black surface and imaged from above using a digital webcam (Creative labs, UK). Experiments were performed at 25 °C. Upon initiation of crawling larvae were recorded for 120 s (0.2 frames s^−1^) using VirtualDub software. Images were analysed using ImageJ. Briefly videos were batch thresholded and a custom macro used to track, via the MTrack2 plugin, and plot the larval positions. These data were then used to determine the mean larval velocity. Videos were manually scored for the number of times each larvae made a distinct directional change.

Genetic interaction eye screens

Genetic interaction screens were performed as described previously (Ahmad et al., 2009; West et al., 2015). Briefly the eye specific driver *GMR*-Gal4 was used to express UAS-*CHMP2B*^*Intron5*^ and UAS-*GCLC* transgenes. Black melanotic spots on the surface of the fly eye were quantified as low (fewer than 15 black spots), medium (15 spots of melanisation but <50% of the eye affected) or high (> 50% of the eye subjected to melanisation).

#### 6.1.4 RNA extraction

Third instar larval brains were collected and immediately snap frozen on dry ice. For each condition (vehicle vs UDCA) and genotype (wildtype vs CHMP2B^Intron5^), five replicates were generated (*n* = 7 brains/replicate). Total RNA was extracted using NucleoSpin RNA extraction kit (Macherey-Nagel, UK), according to the manufacturer's instructions. Purified RNA was quantified using spectrophotometry (NanoDrop; Thermo Scientific, DE, USA) and microfluidic analyzer, Agilent 2100 Bioanalyzer (Agilent Technologies, UK).

#### 6.1.5 RNA libraries and sequencing

mRNA libraries were generated from 1 μg of total RNA using the NEBNext RNA Ultra Directional Library kit for Ilumina in conjunction with the NEBNext Poly(A) mRNA Magnetic Isolation Module (NEB, UK) according to the manufacturer instructions. Libraries were sequenced on an Ilumina® HiSeq3000 (University of Leeds, UK).

#### 6.1.6 RNA-seq analysis

Abundance of *gclc* transcripts were compared between wildtype and pan-neuronal CHMP2B^Intron5^expressing animals. Reads were checked and trimmed using FastQC version 11.0.5 (Wingett and Andrews, 2018) and Cutadapt version 1.8.3 (Bray et al., 2016). Sequence reads were aligned to the *Drosophila melanogaster* FlyBase release 6.20 transcriptome using Salmon 0.6.0 (Patro et al., 2017). Sleuth 0.29.0 was used (Pimentel et al., 2017) for differential expression analysis of the RNA-seq data. A full linear model containing strain and treatment was fitted to the data. In order to look at the effect of the treatment or the strain, the full model was compared to a reduced model. The effect size of the variable was calculated using a Wald test to give a ß-value, in log_2_ units.

#### 6.1.7 Western blotting

For ATG8/LC3 westerns single VNCs from individual third instar *Drosophila* larvae were boiled directly in 2× Laemmli buffer and loaded onto Mini-PROTEAN® TGX™ 4–20% gradient precast gels. For all other experiments *Drosophila* lysates were extracted from ~10–20 third instar larval VNCs using RIPA + protease and phosphatase inhibitors (Roche cOmplete Ultra, Roche PhosSTOP). Samples were boiled inLaemmli loading buffer and 20–40 μg loaded onto Mini-PROTEAN® TGX™ 4–20% gradient precast gels. For quantification, 3 biological replicates were performed per condition and quantification performed using the ImageJ analyse gels function. All samples were normalised relative to the loading control. Primary antibodies used were anti-ATG8/LC3 (Rabbit, 1:2000, Merck Millipore, Cat^#^ ABC974), anti-p53 (Mouse, 1:200, Santa Cruz Biotechnology Cat^#^ sc-74,574, RRID:AB_1249617), anti-Beta Actin (Mouse, 1:180,000, Proteintech Group Cat^#^ 60008–1-Ig, RRID:AB_2289225) and anti-cleaved Dcp-1 (Rabbit, 1:200, Cell Signalling Technology Cat^#^ 9578, RRID:AB_2721060).

#### 6.1.8 Glutathione S-transferase reporter assays

The *GstD1*-GFP endogenous reporter (Sykiotis and Bohmann, 2008) was used to assay Gst activity in control (*GstD1*-GFP/+;*nSyb*-Gal4/+) or CHMP2B^Intron5^ expressing flies (*GstD1*-GFP/UAS-CHMP2B^Intron5^;*nSyb*-Gal4/+). Flies were raised on either vehicle or UDCA, as described previously, and third instar larvae of the correct genotype briefly washed in HL3 (70 mM NaCl, 5 mM KCl, 1 mM CaCl2·2H2O, 10 mM NaHCO3, 5 mM trehalose, 115 mM sucrose and 5 mM BES in dH2O))and placed into individual wells of a 96-well plate containing 200 μl of cold HL3. GFP fluorescence intensity was monitored using a PHERAstar FSX plate reader using 10 × 10 well scanning. Relative fluorescence intensity, normalised for background levels, were reported.

### 6.2 Cell culture

#### 6.2.1 Culture of primary mammalian neurons

Timed-mated female Wistar rats (Charles River UK) (RRID:RGD_737929) were maintained in accordance with the UK Animals (Scientific Procedures) Act (1986). Cortices were dissected from postnatal day 1 (P1) mixed sex rat pups. Animals were euthanised using pentobarbital injection followed by cervical dislocation, according to Home Office UK guidelines. Neuronal cell suspensions were obtained as previously described (Suman et al., 2016) and cultured in Neurobasal medium (21,103,049, Thermo Scientific) supplemented with B27 (50×, 17,504,044, Thermo Scientific), Glucose (35 mM final concentration, A2494001, Thermo Scientific), l-glutamine (1 mM, 25,030,032, Thermo Scientific), Foetal Calf Serum (5%, Mycoplex, PAA), Penicillin (50 u/ml) and Streptomycin (50 μg/ml, 15,140,122, Thermo Scientific) at 7.5 × 10^5^ cells/ml and maintained at 37 °C in 5% CO_2_.

Neurons were transfected at 12 days in vitro (DIV) with Lipofectamine 2000 (11,668,019, Thermo Scientific) in transfection medium (Suman et al., 2016) with either FLAG-tagged CHMP2B^Wildtype^ or CHMP2B^Intron5^ for 5 h. The cDNAs for catalytic (GCLC, MC203908) and modifying (GCLM, MR225622) subunits of GCL were obtained from Origene. For autophagic flux experiments, FLAG-tagged CHMP2B constructs were co-transfected with GFP-mCherry-LC3 (Pankiv et al., 2007). Immediately following transfection, cells were washed and incubated with transfection medium +/− 10 μM UDCA. After 2/3 days, cells were fixed or lysed for biochemical experiments.

#### 6.2.2 Cell survival

After transfection cell viability was assessed using propidium-iodide exclusion assay, as previously described (Lee et al., 2007). Cells were incubated with Propidium iodide for 2 min in accordance with manufacturer's instructions.

#### 6.2.3 MTT assay

MTT assay for cell viability was carried out as previously described (Ugbode et al., 2017). After treatment, primary rat neurons were washed with HBM buffer and incubated with 500 μl of MTT buffer (0.5 mg/ml Thiazoyl Blue Tetrazolium Bromide (Sigma) in HBM) at 37 °C for 1 h. The formazide precipitate was solubilised with 300 μl DMSO per well. 200 μl was transferred to a 96 well plate and absorbance measured using a plate reader (BMG Fluostar λ = 490 nm).

#### 6.2.4 Immunofluorescence and cell imaging

Cells were washed with phosphate buffered saline (PBS) and fixed for 30 min at room temperature (22 °C) with 4% paraformaldehyde (containing 4% sucrose) (Sigma) in PBS as previously described (Ugbode et al., 2014). Cells were permeabilised in 0.5% NP40 in PBS for 5 min at room temperature. Primary antibodies were incubated overnight at 4 °C. Primary antibodies used were anti-FLAG (M2 clone, mouse, Sigma, 1:1000, Cat^#^ F1804, RRID:AB_262044), anti-RAB4a (Clone 4E11, mouse, Santa Cruz Biotechnology (SCBT), 1:200, Cat^#^ SC-517263) anti-RAB5 (Clone C8B1, rabbit, Cell Signalling Technology (CST), 1:200, Cat^#^ 3547, RRID:AB_2300649), anti-RAB7 (Clone B-3, mouse, SCBT, 1:200, Cat^#^ SC-376362, RRID:AB_10987863), anti-ubiquitin (Clone FK2, Enzo Life Science, 1:500, Cat^#^ BML-PW8810, RRID:AB_10541840), anti-GCLC (Clone H-5, SCBT, 1:200, Cat^#^ SC-390811, RRID:AB_2736837) and anti-GFP (Guinea pig, Synaptic Systems, 1:1000, Cat^#^ 132005, RRID:AB_11042617). mCherry was detected using FluoTag-X4, ATTO 542 (1:500, Synaptic Systems, Cat^#^ N0404-At542, RRID:AB_2744638). Corresponding Alexafluor secondary antibodies (1:500, Thermo Scientific) were incubated for 1 h at room temperature before mounting with Fluoromount (Sigma).

#### 6.2.5 Microscopy and image analysis

Images were collected on an inverted Zeiss microscope (880) using 20× and 63× Plan Neofluar objectives using Zeiss filter sets for DAPI and Alexa 488/546/633. Images were taken at an aspect ratio of 2048 × 2048. Images of neurons were traced using the NeuronJ plugin in ImageJ (1.6.0). Individual traces were thresholded and sholl analysis was conducted using the Sholl plugin.

**The following are the supplementary data related to this article.Supplementary Fig. 1:**
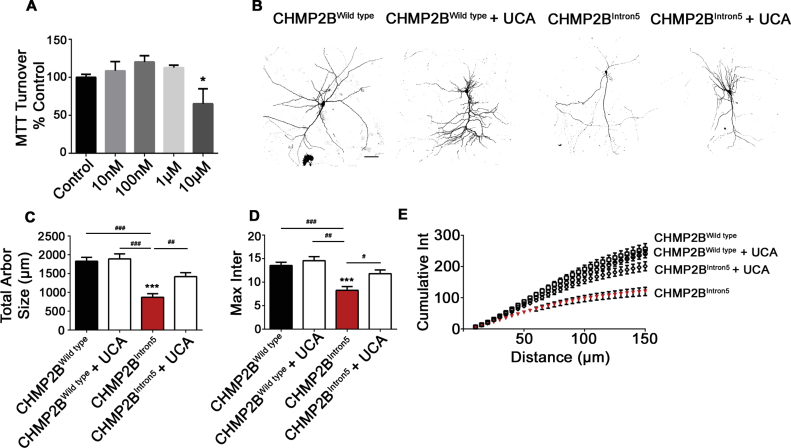
UCA Prevents CHMP2B^Intron5^ Induced Dendritic Loss. A. UCA does not cause mitochondrial toxicity up to 1 μM (48 h) as assessed by MTT turnover. Concentrations of 10 μM UCA significantly reduce MTT absorbance. Data represents mean ± SEM analysed using one-way ANOVA and Bonferroni post hoc test (^⁎^p < .05). Each treatment expressed as percentage of ethanol treated controls (*n* = 3 biological replicates). B. Representative micrographs of mature neurons expressing FLAG-tagged CHMP2B^Wildtype^ or CHMP2B^Intron5^ ± UCA (1 μM, 48 h). Scale bar = 50 μm. C-E. Quantification of total arbour size (C) total number of intersections (D) and cumulative number of intersections per unit distance (E) in CHMP2B^Wildtype^ or CHMP2B^Intron5^ ± UCA (1 μM, 48 h). Data represents mean ± SEM analysed using one-way ANOVA and both Bonferroni (^⁎⁎⁎^p < .001) and Tukey's multiple comparisons post hoc test (^##^p < .01, ^###^p < .001). 35 neurons analysed, per condition, across 3 biological replicates.

Supplementary Table 1 RNA-seq dataset of Drosophila wildtype versus CHMP2B^Intron5^ in neurons treated with vehicle or UDCA. Wildtype or Drosophila expressing CHMP2B^Intron5^ in neurons were fed with 600 μM UDCA or vehicle. At third instar larval stage brains were dissected and processed for RNAseq analysis. *N* = 7brains/replicate. Supplementary Table 1
